# Body mass index related to executive function and hippocampal subregion volume in subjective cognitive decline

**DOI:** 10.3389/fnagi.2022.905035

**Published:** 2022-08-17

**Authors:** Ruilin Chen, Guiyan Cai, Shurui Xu, Qianqian Sun, Jia Luo, Yajun Wang, Ming Li, Hui Lin, Jiao Liu

**Affiliations:** ^1^National-Local Joint Engineering Research Center of Rehabilitation Medicine Technology, Fujian University of Traditional Chinese Medicine, Fuzhou, China; ^2^College of Rehabilitation Medicine, Fujian University of Traditional Chinese Medicine, Fuzhou, China; ^3^Affiliated Rehabilitation Hospital, Fujian University of Traditional Chinese Medicine, Fuzhou, China; ^4^Department of Physical Education, Fujian University of Traditional Chinese Medicine, Fuzhou, China; ^5^Fujian Key Laboratory of Rehabilitation Technology, Fuzhou, China; ^6^Traditional Chinese Medicine Rehabilitation Research Center of State Administration of Traditional Chinese Medicine, Fujian University of Traditional Chinese Medicine, Fuzhou, China; ^7^Key Laboratory of Orthopedics and Traumatology of Traditional Chinese Medicine and Rehabilitation, Ministry of Education, Fujian University of Traditional Chinese Medicine, Fuzhou, China

**Keywords:** subjective cognitive decline, executive function, inhibition control function, hippocampal subregion, CA1

## Abstract

**Objective:**

This study aims to explore whether body mass index (BMI) level affects the executive function and hippocampal subregion volume of subjective cognitive decline (SCD).

**Materials and methods:**

A total of 111 participants were included in the analysis, including SCD (38 of normal BMI, 27 of overweight and obesity) and normal cognitive control (NC) (29 of normal BMI, 17 of overweight and obesity). All subjects underwent the Chinese version of the Stroop Color-Word Test (SCWT) to measure the executive function and a high-resolution 3D T1 structural image acquisition. Two-way ANOVA was used to examine the differences in executive function and gray matter volume in hippocampal subregions under different BMI levels between the SCD and NC.

**Result:**

The subdimensions of executive function in which different BMI levels interact with SCD and NC include inhibition control function [SCWT C-B reaction time(s): *F*_(1,104)_ = 5.732, *p* = 0.018], and the hippocampal subregion volume of CA1 [*F*_(1,99)_ = 8.607, *p* = 0.004], hippocampal tail [*F*_(1,99)_ = 4.077, *p* = 0.046], and molecular layer [*F*_(1,99)_ = 6.309, *p* = 0.014]. After correction by Bonferroni method, the population × BMI interaction only had a significant effect on the CA1 (*p* = 0.004). Further analysis found that the SCWT C-B reaction time of SCD was significantly longer than NC no matter whether it is at the normal BMI level [*F*_(1,104)_ = 4.325, *p* = 0.040] or the high BMI level [*F*_(1,104)_ = 21.530, *p* < 0.001], and the inhibitory control function of SCD was worse than that of NC. In the normal BMI group, gray matter volume in the hippocampal subregion (CA1) of SCD was significantly smaller than that of NC [*F*_(1,99)_ = 4.938, *p* = 0.029]. For patients with SCD, the high BMI group had worse inhibitory control function [*F*_(1,104)_ = 13.499, *p* < 0.001] and greater CA1 volume compared with the normal BMI group [*F*_(1,99)_ = 7.619, *p* = 0.007].

**Conclusion:**

The BMI level is related to the inhibition control function and the gray matter volume of CA1 subregion in SCD. Overweight seems to increase the gray matter volume of CA1 in the elderly with SCD, but it is not enough to compensate for the damage to executive function caused by the disease. These data provide new insights into the relationship between BMI level and executive function of SCD from the perspective of imaging.

## Introduction

Subjective cognitive decline (SCD) refers to the decline in subjective memory or cognitive function, but there is no obvious cognitive dysfunction and no obvious impairment of daily living ability in objective behavioral examination (Jessen et al., 2014). SCD is a state between normal aging and mild cognitive impairment (MCI), which is considered to be one of the most initial cognitive change in the pathogenesis of Alzheimer’s disease (AD) (Jessen et al., 2020). A recent study found that the prevalence of SCD in the elderly population > 50 years was 26.6% (Liew, 2019), and SCD increased the risk of progression to MCI in the elderly by 1.73 times and the risk of progression to AD by 1.9 times (Pike et al., 2021).

One variable that may play an important role in the development of AD is obesity, which is associated with numerous deleterious health conditions (Mallorquí-Bagué et al., 2018; Piché et al., 2020) including late-life dementia (Kivipelto et al., 2018). Body mass index (BMI), one measure of obesity, has a complex relationship with cognitive function in the elderly. Previous studies found that the cognitive dimensions of BMI’s impact are different across clinical stages of AD. For instance, in dementia or MCI stage of AD, a higher BMI is related to the worse overall cognitive function, memory, attention, and executive function in the elderly (Calderón-Garcidueñas et al., 2019; Sanchez-Flack et al., 2021). In the elderly population with normal cognitive status, higher BMI predicts worse executive function (Gunstad et al., 2007; Beyer et al., 2017), while BMI is not significantly associated with attention and memory dimensions (Schmeidler et al., 2019). These studies suggested that different from other cognitive dimensions the effects of BMI on executive function may appear to be present throughout different clinical stages of AD. However, the relationship between BMI and executive function in SCD (an early stage of AD) is unclear. In addition, our previous preliminary study found that overweight and obese patients with SCD had a worse executive function compared with patients with SCD in the normal weight group (Xu et al., 2021). However, a previous study lacked further investigation in normal controls (NCs) to check the interaction of BMI level and disease on executive function in patients with SCD. In addition, the underlying mechanism is also unclear.

Neuroimaging studies have suggested early AD-like structural brain alterations in SCD (Pini and Wennberg, 2021). The hippocampus plays a critical role in cognition (Lisman et al., 2017). A previous study found that SCD exhibits a consistent pattern of hippocampal atrophy (Chen et al., 2021). Some studies have observed a decreased hippocampal volume in individuals with SCD both at baseline and during a significant longitudinal decline (Scheef et al., 2012; Sánchez-Benavides et al., 2018; van Rooden et al., 2018; Yue et al., 2018), with an annual decrease of 1.9% (Cherbuin et al., 2015; Wang et al., 2020). The hippocampus is composed of multiple subregions such as the dentate gyrus (DG), cornu ammonis (CA) region, and subiculum (SUB), all of which play specific roles in the circuits of the hippocampus (O’Keefe et al., 2007). For example, the CA1 subregion and the entorhinal cortex can represent a variety of different information (time, space, etc.), and the SUB, as a transition region between the two subregions, can accept the direct input of synapses in CA1 subregion and project it to different cortex and subcortical regions (Matsumoto et al., 2019); CA1, CA2/3, and DG play complementary roles in supporting episodic memory by allowing one to remember specific items, as well as their relationships, within a shared context (Dimsdale-Zucker et al., 2018). With the progression of AD, the volume of hippocampal subregions shows an obvious decreasing trend (Zhao et al., 2019). Studies have found that in people with risk of AD smaller volumes of the hippocampal fimbria, presubiculum, and SUB showed the associations with poor performance on executive function (Evans et al., 2018). While in patients with MCI, smaller hippocampal subregion (CA1) volume is associated with worse executive function (Suo et al., 2017). However, the relationship between the hippocampal subregion and the executive function subdimension in SCD is still not clear.

It is worth mentioning that the hippocampus is a key structure involved in body weight regulation (Davidson et al., 2007; Kanoski and Grill, 2017). Neuroimaging studies incorporating structural magnetic resonance imaging (MRI) reported that patients with AD with higher BMI levels have a smaller hippocampal volume (Ly et al., 2021). However, the relationship between BMI and cognitive function/hippocampal volume in different stages of AD is inconsistent. Kivimäki et al. (2018) reported that when BMI was assessed > 20 years before the diagnosis of dementia, a higher BMI was associated with an increased risk of dementia, whereas when BMI was assessed < 10 years before the diagnosis, a lower BMI predicted dementia. In addition, studies reported that overweight/obesity was positively correlated with the hippocampal volume of subjects (Widya et al., 2011; Ma et al., 2019). Animal experiments show that obesity affects the hippocampal subregion (CA1, CA3) of rats with pre-AD and MCI models (Ivanova et al., 2020). However, it is unclear what the effects are of different BMIs on the volume of the hippocampal subregion in the elderly with SCD and whether there is an interactive effect between the BMI and disease condition on the volume of hippocampal subregions.

This study aims to compare the difference in executive function of the elderly SCD and NC with different BMI levels and explore whether there are differences in hippocampal subregions related to BMI levels in different cognitive states. We hypothesized that the effect of BMI level on executive function in patients with SCD may be related especially to the hippocampal subregion gray matter volume.

## Materials and methods

### Participants

In this study, we recruited 111 elderly subjects aged > 60 years, who voluntarily participated in a free questionnaire survey and physical examination in communities in Fuzhou, Fujian Province, including 65 elderly people with SCD and 46 elderly NC. The SCD and NC participants were divided into normal BMI group and overweight/obesity (high BMI) group according to the Chinese adult overweight and obesity prevention and control guidelines (China Obesity Working Group, 2004) (normal weight, BMI between 18.5 and 23.9 kg/m^2^; overweight and obese, BMI ≥ 24.0 kg/m^2^). This study was approved by the Medical Ethics Committee of the Affiliated Rehabilitation Hospital of Fujian University of Traditional Chinese Medicine. All participants signed an informed consent form before taking the tests.

Inclusion criteria of SCD included (1) meeting the SCD conceptual framework proposed by Jessen et al. (2014) and China AD Preclinical Alliance (Ying Han, 2018); (2) aged 60–75 years; (3) BMI ≥ 18.5 kg/m^2^; and (4) informed consent, voluntary participation. The SCD conceptual framework was as follows: (1) subjective decline in memory rather than other domains of cognition; (2) onset of SCD within the last 5 years; (3) age at SCD onset of at least 60 years; (4) worries associated with SCD; (5) worse self-perceived memory than others in the same age group; and (6) absence of objective clinical impairment of MCI, Montreal Cognitive Assessment (MoCA) (Nasreddine et al., 2005) total score ≥ 26 (Langa and Levine, 2014).

Normal cognitive control inclusion criteria included (1) normal activities of daily living; (2) no self-SCD and no obvious memory impairment; (3) normal cognitive testing (MoCA score ≥ 26 points); (4) no significant behavioral and language impairments; (5) aged 60–75 years; (6) BMI ≥ 18.5 kg/m^2^; and (7) informed consent, voluntary participation.

Subjective cognitive decline and NC exclusion criteria included (1) hypertensive patients with uncontrolled blood pressure; (2) history of alcohol and drug abuse; (3) severe anxiety and depression, as indicated by the Hamilton Depression Scale (HAMD) (Hamilton, 1960) score > 24 points or Hamilton Anxiety Scale (HAMA) (Hamilton, 1959) score > 29 points; (4) decline in cognitive function caused by other diseases (such as mental diseases and poisoning); and (5) unable to cooperate with the tester due to other physiological and psychological reasons.

### Clinical assessment

In the form of a questionnaire, the basic demographic data of the subjects (age, gender, BMI, years of education) and medical history (hypertension, hyperlipidemia, type II diabetes mellitus [T2DM], etc.), as well as medication history, were recorded in detail by professionally trained assessors. Medication history refers to the last 3-month routine medication self-reported by subjects when receiving the questionnaire of this study. We mainly recorded the use of drugs that control hypertension, hyperlipidemia, and T2DM. In addition, any other medication the participant used within the last 3 months was also recorded.

### Neuropsychological assessment

Montreal Cognitive Assessment was used to assess the global cognitive function of subjects, with a total score of 30 points (the higher the score, the better the global cognitive function). HAMD and HAMA were used to assess the severity of depression and anxiety.

The Stroop Color-Word Test (SCWT) (Golden and Golden, 1981) evaluates the executive function. The SCWT version (Xu et al., 2021) adopted in this study consists of three cards, each with 24 characters. SCWT A is composed of red, yellow, blue, or green dots; SCWT B is composed of Chinese characters printed in the same color (red, yellow, blue, or green, the color of the characters is consistent with the meaning of the word); and SCWT C prints four kinds of Chinese characters with different colors (red, yellow, blue, or green, the color of the characters is inconsistent with the meaning of the word, such as “yellow” printed in red color). The longer the response time of each card, the worse the execution function (Wecker et al., 2000; Homack and Riccio, 2004). The analysis indicators are the reaction time of each card and the SCWT C-B reaction time. A larger SCWT C-B reaction time represents greater interference from conflicting response sets or poorer inhibitory control (Scarpina and Tagini, 2017; Rabi et al., 2020).

### Brain imaging acquisition

The brain MRI data were acquired on a 3.0 T Prisma scanner system (Siemens Medical Solutions, Erlangen, Germany) with a 64–channel head coil. Before MRI scanning, inform the precautions of MRI scanning again and sign the consent form for MRI scanning. Confirm that the subjects have no scanning contraindications such as metal implants and claustrophobia. Ask the subjects to stay still during the scanning process, use rubber earplugs and soft head pads to reduce noise, and fix the position of the head. If they feel uncomfortable, press the alarm in their hand to instruct the staff to stop the scanning. The T1–MPRAGE images were collected using the following parameters: field of view, 256 mm * 256 mm; repetition time, 2,300 ms; echo time, 2.94 ms; flip angle, 15°; slice thickness, 1 mm; and slices, 160. In addition, all subjects in this study were screened with an appropriate MRI scan (T2-weighted sequence) to check the vascular injuries such as stroke and brain tumor before initiation of the study. None of the eligible subjects included had obvious vascular lesions.

### Brain imaging processing

The T1 image data were preprocessed using the MRIconvert (a toolbox for image data conversion, version 2.0 Rev. 235^1^) and FreeSurfer software (a toolbox for image data analysis, version 7.1.0^2^) under the Lunix system.

The FreeSurfer 7.1.0 software was used to extract the hippocampal volume in the subcortical nucleus segmentation file of each subject generated by pretreatment. During segmentation, the image of each subject is first converted from the individual space to the FreeSurfer standard space to ensure accurate segmentation, and then the hippocampus of each subject is divided into 19 regions according to the hippocampal subregion segmentation template on the official website of FreeSurfer.^3^ According to Supplementary Table 1, the subdivided 19 regions were combined into hippocampal tail, hippocampal fissure, fimbria, parasubiculum, subiculum, presubiculum, CA1, CA3, CA4, molecular layer, granule cell layer, and molecular layer of the dentate gyrus (GC-ML-DG) and hippocampal amygdala transition area (HATA), the 12 hippocampal subregions, and CA2 is always included in CA3 (Fischl, 2012; Iglesias et al., 2015; Supplementary Figure 1).

## Statistical analysis

### Behavioral data analysis

Analysis was performed using the SPSS software (version 26.0 for Windows, IL, United States). The categorical variables are expressed by the number of cases *n* (%), and the comparison between groups was performed using chi-square test. The continuous variables are expressed as mean ± SD, and the comparison between groups was performed using one-way ANOVA. If *p* < 0.05, the difference was considered to be statistically significant, and the *post hoc* comparison was performed using Fisher’s least significant difference method.

Two-way ANOVA was used for the comparison between groups of SCWT test (the first factor was population grouping and the second factor was BMI grouping, to explore the main effects of population grouping and BMI grouping and the interaction between them). Age, gender, and years of education were used as covariates; *p* < 0.05 was considered to be statistically significant. When there is an interaction between population grouping and BMI grouping, simple main effect and paired comparative analysis were used to further study whether different populations and different BMI improve or reduce executive function.

### Brain imaging analysis

The volume of each subject’s hippocampal subregion was extracted and analyzed by two-way ANOVA based on region of interest (ROI) level (the first factor is population grouping and the second factor is BMI grouping) with the total hippocampal volume, intracranial volume, age/gender/years of education/hypertension/hyperlipidemia/T2DM as covariates. The brain regions with interactive gray matter volume differences were obtained, and the interaction effects between them were further analyzed using the SPSS 26.0 software. When there is an interaction between population grouping and BMI grouping, simple main effect and paired comparative analysis were used to further study whether different populations and different BMI increase or decrease the gray matter volume of hippocampal subregions. For the brain area with significant interaction, the association between the gray matter volume and the corresponding score of SCWT test was done using the partial correlation analysis with age, gender, and years of education as covariates. Multiple comparisons were corrected using Bonferroni method.

## Results

### Demographic characteristics

The comparison of general demographic data and personal medical history of each group (i.e., NC-normal BMI, NC-high BMI, SCD-normal BMI, SCD-high BMI) is shown in Table 1. The results of intergroup comparison showed that no significant group difference was found among the general demographic data of the four groups except BMI. After adjusting for age, gender, and years of education, the mean MoCA score of subjects with SCD included in this study was 27.40 ± 1.07 ≥ 26, which suggested that there was no obvious objective index abnormality.

**TABLE 1:** Demographics, disease medical history, and cognitive features of participants.

	NC		SCD		*F/Z*/χ^2^	*P*-value
				
	Normal BMI (*n* = 29)	High BMI (*n* = 17)	Normal BMI (*n* = 38)	High BMI (*n* = 27)		
Age (years)^a^	66.24 ± 4.57	68.53 ± 4.05	65.39 ± 4.72	65.85 ± 4.61	6.459	0.091
Gender [*n* (%)]^b^					7.184	0.066
Male	6 (20.7)	6 (35.3)	10 (26.3)	28 (73.7)		
Female	23 (79.3)	11 (64.7)	28 (73.7)	13 (48.1)		
BMI (kg/m^2^)^a^	21.60 ± 1.64	26.23 ± 1.76	21.81 ± 1.37	25.90 ± 2.64	79.395	<0.001
Education (years)^a^	11.12 ± 2.45	11.56 ± 2.32	12.54 ± 2.81	12.28 ± 2.58	1.916	0.131
Hypertension [*n* (%)]^b^	7 (24.1)	9 (52.9)	14 (36.8)	14 (51.9)	5.977	0.113
Hyperlipidemia [*n* (%)]^b^	5 (17.2)	5 (29.4)	5 (13.2)	5 (18.5)	2.118	0.548
T2DM [*n* (%)]^b^	5 (17.2)	3 (17.6)	9 (23.7)	6 (22.2)	0.552	0.907
Medicine [*n* (%)]^b^	13 (44.8)	9 (52.9)	20 (52.6)	17 (63.0)	1.855	0.603
MoCA score^a^	26.97 ± 1.05	27.59 ± 1.12	27.53 ± 1.03	27.22 ± 1.12	7.045	0.070
HAMD score^a^	2.55 ± 2.10	1.94 ± 2.14	3.47 ± 2.55	2.26 ± 2.18	6.548	0.088
HAMA score^a^	3.28 ± 2.58	2.76 ± 1.95	3.00 ± 1.79	3.04 ± 1.48	1.042	0.791

^a^Continuous variable, one-way ANOVA is adopted, and mean ± SD is used for statistical description.

^b^Categorical variables were statistically described by chi-square test, and n (%) is used for statistical description.

BMI, body mass index; T2DM, type II diabetes mellitus; MoCA, the Montreal Cognitive Assessment; HAMD, the Hamilton Depression Scale; HAMA, the Hamilton Anxiety Scale. Medicine: medication history refers to the last 3-month routine medication self-reported by subjects when receiving the questionnaire of this study, and mainly records the use of drugs that control hypertension, hyperlipidemia, and T2DM. In addition, any other medications the participant has used within the last 3 months were also recorded.

### Neuropsychological characteristics

Montreal Cognitive Assessment score, HAMD score, and HAMA score of each group (NC-normal BMI, NC-high BMI, SCD-normal BMI, and SCD-high BMI) are compared in Table 1. The results of intergroup comparison showed that there was no significant difference in MOCA score, HAMD score, and HAMA score among the four groups (*p* > 0.05).

The comparison of SCWT scores of each group is shown in Table 2. We observed significant interaction effects for SCWT C reaction time (s) [*F*_(1,104)_ = 8.017, *p* = 0.006, partial η^2^ = 0.072] and SCWT C-B reaction time (s) [*F*_(1,104)_ = 5.732, *p* = 0.018, partial η^2^ = 0.052], as well as significant population main effects [SCWT C reaction time (s): *F*_(1,104)_ = 32.745, *p* < 0.001; SCWT C-B reaction time (s): *F*_(1,104)_ = 23.411, *p* < 0.001] and BMI main effects [SCWT C reaction time (s): *F*_(1,104)_ = 7.593, *p* = 0.007; SCWT C-B reaction time (s): *F*_(1,104)_ = 5.33, *p* = 0.023], respectively.

**TABLE 2:** Comparison of SCWT response time of participants.

	NC		SCD		*F*	interaction *P*-value	η*^2^*
					
	Normal BMI (*n* = 29)	High BMI (*n* = 17)	Normal BMI (*n* = 38)	High BMI (*n* = 27)			
SCWT A reaction time(s)^a^	15.72 ± 3.24	15.05 ± 3.56	18.61 ± 4.42	20.97 ± 6.46	1.954	0.165	0.018
SCWT B reaction time(s)^a^	19.42 ± 4.65	20.01 ± 4.96	22.26 ± 5.34	28.82 ± 13.08	3.163	0.078	0.030
SCWT C reaction time(s)^a,b^	33.01 ± 9.22	34.96 ± 10.28	38.88 ± 12.80	53.67 ± 13.22	8.017	0.006	0.072
SCWT C-B reaction time(s)^a,b^	13.59 ± 7.63	14.95 ± 8.15	17.73 ± 10.83	28.65 ± 13.31	5.732	0.018	0.052

^a^Continuous variable, two-way ANOVA is adopted, and mean ± SD is used for statistical description; the unit is seconds.

^b^There is an interaction between population grouping and BMI grouping after correction by Bonferroni method. SCWT, the Stroop Color-Word Test.

The two-way ANOVA was carried out with age, gender, and years of education as covariates.

*Post hoc* analysis showed that the SCWT C reaction time (s) and SCWT C-B reaction time (s) of the SCD-normal BMI group were significantly larger than the NC-normal BMI group, which indicates that the inhibitory control function of the SCD-normal BMI group was worse than the NC-normal BMI group [SCWT C reaction time (s): *F*_(1,104)_ = 6.050, *p* = 0.016; SCWT C-B reaction time (s): *F*_(1,104)_ = 4.325, *p* = 0.040]. The SCWT C-B reaction time (s) of SCD-high BMI group was significantly higher than the NC-high BMI group, indicating that the inhibitory control function of the SCD-high BMI group was worse than the NC-high BMI group [SCWT C reaction time (s): *F*_(1,104)_ = 30.115, *p* < 0.001; SCWT C-B reaction time (s): *F*_(1,104)_ = 21.530, *p* < 0.001]. The SCWT C reaction time (s) and SCWT C-B reaction time (s) of the SCD-normal BMI group were significantly smaller than the SCD-high BMI group, suggesting that the inhibitory control function of the SCD-normal BMI group was better than those of the SCD-high BMI group [SCWT C reaction time (s): *F*_(1,104)_ = 19.053, *p* < 0.001; SCWT C-B reaction time (s): *F*_(1,104)_ = 13.499, *p* < 0.001]. There was no significant difference in the inhibitory control function [SCWT C reaction time (s): *p* = 0.982 > 0.05; SCWT C-B reaction time (s): *p* = 0.973 > 0.05] between the NC-normal BMI group and the NC-high BMI group. The results of SCWT C reaction time (s) and SCWT C-B reaction time (s) are given in Tables 2, 3.

**TABLE 3:** *Post hoc* analysis of SCWT tests with interactions.

		Normal BMI	High BMI	NC	SCD
SCWT C reaction time(s)^a^	***F***	6.050	30.115	0.001	19.053
	***P***	0.016	<0.001	0.982	<0.001
SCWT C-B reaction time(s)^a^	***F***	4.325	21.530	0.001	13.499
	***P***	0.040	<0.001	0.973	<0.001

^a^There is an interaction between population grouping and BMI grouping. SCWT, the Stroop Color-Word Test.

The two-way ANOVA was carried out with age, gender, and years of education as covariates.

For SCWT A reaction time (s) and SCWT B reaction time (s), we did not find significant differences in the interaction terms between population grouping and BMI grouping (*p* > 0.05; see Table 2).

### Brain imaging characteristics

A comparison of hippocampal subregion volume among the four groups is shown in Table 4. ANOVA showed that the population × BMI interaction had a significant effect on CA1 [*F*_(1,99)_ = 8.607, *p* = 0.004, partial η^2^ = 0.080], molecular layer [*F*_(1,99)_ = 6.309, *p* = 0.014, partial η^2^ = 0.060], and hippocampal tail [*F*_(1,99)_ = 4.077, *p* = 0.046, partial η^2^ = 0.040]. *Post hoc* analysis showed that compared with SCD-normal BMI, CA1 [*F*_(1,99)_ = 7.619, *p* = 0.007], molecular layer [*F*_(1,99)_ = 6.351, *p* = 0.013], and hippocampal tail [*F*_(1,99)_ = 4.481, *p* = 0.037] volumes were larger in the SCD-high BMI group. The volume of CA1 [*F*_(1,99)_ = 4.938, *p* = 0.029] was smaller in the SCD-normal BMI group compared with the NC-normal BMI group. For other hippocampal subregions (hippocampal tail, hippocampal fissure, fimbria, parasubiculum, subiculum, presubiculum, CA3, CA4, GC-ML-DG, HATA), we did not find significant differences in the interaction term between population grouping and BMI grouping (*p* > 0.05; see Table 5).

**TABLE 4:** Comparison of the gray matter volume of hippocampal subregions.

	NC		SCD		*F*	interaction *P*-value	η^2^
					
	Normal BMI (*n* = 29)	High BMI (*n* = 17)	Normal BMI (*n* = 38)	High BMI (*n* = 27)			
Fimbria^a^	140.02 ± 27.68	136.88 ± 42.62	140.71 ± 42.62	135.30 ± 30.08	0.468	0.496	0.005
Hippocampal tail^a^	1131.37 ± 148.57	1124.49 ± 145.31	1211.41 ± 145.31	1174.71 ± 150.21	4.077	0.046	0.040
Hippocampal fissure^a^	311.08 ± 57.45	332.80 ± 43.91	313.00 ± 43.91	334.24 ± 61.54	0.085	0.771	0.001
Parasubiculum^a^	118.63 ± 22.72	128.19 ± 39.14	125.66 ± 39.14	129.06 ± 26.69	0.565	0.454	0.006
HATA^a^	372.18 ± 42.04	365.76 ± 61.01	385.50 ± 61.01	383.93 ± 47.42	0.356	0.552	0.004
Subiculum^a^	875.96 ± 88.15	864.50 ± 108.60	900.03 ± 108.6	911.20 ± 99.37	0.002	0.961	0.000
Presubiculum^a^	578.68 ± 61.11	587.29 ± 91.56	603.47 ± 91.56	599.56 ± 72.49	1.342	0.249	0.013
CA1^a,b^	1261.17 ± 173.97	1191.34 ± 173.97	1229.23 ± 149.79	1319.61 ± 157.07	8.607	0.004	0.080
CA3^a^	395.54 ± 58.86	390.07 ± 68.34	414.46 ± 56.01	433.96 ± 57.62	0.479	0.490	0.005
CA4^a^	464.64 ± 52.40	459.88 ± 64.73	489.89 ± 48.30	502.93 ± 53.16	0.256	0.614	0.003
Molecular layer^a^	1069.42 ± 112.25	1045.28 ± 149.39	1107.70 ± 117.61	1139.04 ± 124.48	6.309	0.014	0.060
GC-ML-DG^a^	535.52 ± 62.95	524.13 ± 78.41	562.64 ± 60.00	576.00 ± 67.07	0.723	0.397	0.007

^a^Continuous variable, two-way ANOVA is adopted, and mean ± SD is used for statistical description; the unit of volume is cubic millimeter.

^b^There is an interaction between population grouping and BMI grouping after correction by Bonferroni method.

HATA, hippocampal amygdala transition area; CA, cornu ammonis; GC-ML-DG, granule cell layer and molecular layer of the dentate gyrus. The two-way ANOVA was carried out with age, gender, years of education, hypertension, hyperlipidemia, type II diabetes mellitus, whole hippocampus volume, and intracranial volume as covariates.

**TABLE 5:** *Post hoc* analysis of hippocampal subregions with interactions.

		Normal BMI	High BMI	NC	SCD
CA1^a,b^	***F***	4.938	3.500	2.140	7.619
	***P***	0.029	0.064	0.147	0.007
Molecular layer^a^	***F***	2.726	3.253	1.260	6.351
	***P***	0.102	0.074	0.264	0.013
Hippocampal tail^a^	***F***	3.113	1.208	0.684	4.481
	***P***	0.818	0.274	0.410	0.037

^a^There is an interaction between population grouping and BMI grouping before correction by Bonferroni method.

^b^There is an interaction between population grouping and BMI grouping after correction by Bonferroni method.

The two-way ANOVA was carried out with age, gender, years of education, hypertension, hyperlipidemia, type II diabetes mellitus, whole hippocampus volume, and intracranial volume as covariates.

**FIGURE 1:**
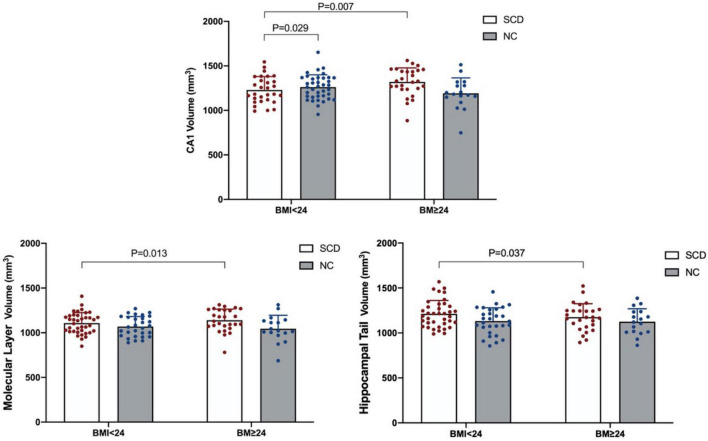
Significant population × BMI interaction effect on gray matter volume in the hippocampal subregion.

After correction by Bonferroni’s method, ANOVA showed that the population × BMI interaction had a significant effect on CA1. *Post hoc* analysis showed that in the normal BMI group gray matter volume was decreased in CA1 of SCD compared with NC; in the SCD population, gray matter volume of CA1 in the high BMI group was significantly increased compared with normal BMI (see Figure 1). In the NC population, we did not observe any significant effect of changes in BMI level on CA1 gray matter volume (*p* > 0.05). In the high BMI population, we did not observe any significant difference in CA1 gray matter volume between the SCD and NC population (*p* > 0.05).

After correction by the Bonferroni method, we did not find significant correlation between gray matter volume changes in CA1 and SCWT C reaction time (s) (*p* > 0.05/4) or CA1 and SCWT C-B reaction time (s) (*p* > 0.05/4; see Supplementary Tables 2, 3).

## Discussion

This study compared the effects of different BMI levels (high BMI and normal BMI) and different populations (SCD and NC) on executive function and hippocampal subregion volume. Our results showed significant interaction effects between the population group and BMI group in the executive function (inhibitory control function) and hippocampal subregion (CA1) gray matter volume. In addition, we found significant main effects in the BMI group and population group. Our results suggested that SCD has a worse executive function (inhibitory control function) than NC regardless of the BMI level, but overweight and obesity aggravate the degree of impairment of executive function in the elderly with SCD. For the normal BMI subgroup, the CA1 volume of SCD was smaller than that of NC. Furthermore, we found that in the SCD population the CA1 gray matter volume of high BMI was larger than that of normal BMI.

The present results suggest that SCD has a worse executive function (inhibitory control function) than NC in both overweight/obese and normal weight, which is partly consistent with previous studies. A study from Spain (López-Higes et al., 2017) showed that the executive function test (use the Stroop interference index to test) performance scores in the elderly with SCD were lower than healthy controls. This study found that the worse executive function of SCD compared with NC is mainly manifested in inhibitory control function. Although repeating the results of previous studies, this study considered the effect of BMI on patients with SCD and found that elevated BMI aggravated the impairment of inhibitory control function in patients with SCD. A meta-analysis (Yang Y. et al., 2018) showed that individuals who are overweight have deficits in working memory and inhibitory control function. Inhibitory control is an important subdimension of executive function (Dempster, 1992; Bjorklund and Harnishfeger, 1995). The impairment of inhibitory control functions has been identified as the most affected function in the subdomain of MCI executive function (Traykov et al., 2007; Brandt et al., 2009; Johns et al., 2012). These results may be explained from a neurophysiological perspective. Inhibitory-controlled behavior was found to be electrophysiologically correlated in patients with MCI, and neurocognitive mechanisms associated with response inhibition (No Go P300) were impaired in patients with MCI compared with healthy controls (López Zunini et al., 2016). In the preclinical stage of MCI, SCD may also have the same neurocognitive impairment as MCI, so we observed worse inhibitory control function in SCD compared with healthy subjects. As for the impact of BMI on the executive function of SCD, studies have shown that overweight or obesity can significantly reduce brain blood flow, lead to insufficient cerebral perfusion, with the brain lacking enough oxygen and nutrients for a long time, which is the early mechanism of AD (Knight et al., 2021), and is also related to worse executive function (Alosco et al., 2012). This may explain why the increase in BMI leads to the aggravation of executive function impairment in patients with SCD.

In addition, we also found a significant interaction between population grouping and BMI grouping in the hippocampal subregion (CA1), and the hippocampal subregion of CA1 significant atrophy in SCD compared with NC. A recent study (Worker et al., 2018) showed that patients with AD experience greater hippocampal subregional atrophy over time compared to NC subjects, including CA1, molecular layer, CA3, hippocampal tail, fissure, and presubiculum, among which CA1 and molecular layer is more obvious. We also found atrophy in the molecular layer of the hippocampus in SCD before Bonferroni correction, which is partly consistent with a previous study. The results seem plausible as the most distinctive AD-related neuron loss was seen in the CA1 region of the hippocampus, and the neuronal loss in CA1 is not an age-related phenomenon but rather characterizes an overt AD process (West et al., 1994). Some studies considered a sequential pattern of atrophy starting within entorhinal and transentorhinal areas and moving to CA1 and eventually other hippocampus subregions (Csernansky et al., 2005; Apostolova et al., 2010). SCD showed a similar pattern of volume atrophy in the hippocampal subregion as AD, preferentially and mainly involving the CA1 region (Perrotin et al., 2015). These findings may be related to the unique structure and function of hippocampal CA1. The CA1 region of the hippocampus maintains its neuroplastic flexibility well into adulthood and plays an important role in external and internal demands to serve cognitive processes (Walhovd et al., 2014b). However, studies have found that brain regions with high neuroplasticity are more prone to neurodegeneration (Neill, 2012; Bufill et al., 2013). The ability of CA1 may increase its vulnerability to neurotoxic effects, ultimately leading to structural atrophy and functional decline (Walhovd et al., 2014a; Nemeth et al., 2017). Deposition of Aβ occurs in the neocortex and hippocampus many years before the onset of clinical symptoms of AD (Sadigh-Eteghad et al., 2015), while the CA1 area is highly sensitive to pathological changes (Pluta et al., 2021). We therefore speculate that the loss of CA1 volume in subjects with SCD may be significantly associated with neuroplasticity in the hippocampal CA1 region.

It is worth mentioning that the result of this study also showed that the hippocampal subregion (CA1) volume of high BMI index in SCD is significantly higher than that of normal BMI population. A recent longitudinal imaging study (Sun et al., 2020) found that higher BMI in AD populations was associated with larger hippocampus volumes, which is partly consistent with the present result. Sun et al. (2020) pointed out that subjects with higher BMI showed a significant lower Aβ load using PET imaging. Previous studies have suggested that Aβ peptide deposition triggers tau hyperphosphorylation and aggregation in the form of neurofibrillary tangles, and these aggregates lead to inflammation, synaptic damage, neuronal loss, and thus decrease the brain volume (Nelson et al., 2012; He et al., 2018; Vogel et al., 2020; Roda et al., 2022). The accumulation of Aβ pathology enhances hippocampal atrophy in pre-AD (Gordon et al., 2016; Wang et al., 2016). In addition, in contrast to previous study, we found that the increased hippocampal volume caused by the increase in BMI is mainly in the CA1 area. A previous study found that medium and large Aβ plaques are significantly more numerous in CA1 than in other hippocampal subregions (Ugolini et al., 2018), and CA1 may be the most vulnerable region of the hippocampus to neuronal loss (Padurariu et al., 2012; Yang X. et al., 2018). We therefore reasoned that the increased CA1 volume in high-BMI subjects with SCD might be partly mediated by obesity reducing the accumulation of Aβ in the hippocampus.

However, the compensatory increase in CA1 volume in patients with SCD in this study does not appear to be sufficient to compensate for the impairment of executive function in patients with SCD, which is manifested by worse performance on executive function tests in patients with SCD compared with NC. Recent studies have shown that the hippocampus plays an important role in appetite and weight regulation (Alosco et al., 2017; Hsu et al., 2018; Li et al., 2021), and is crucial in the mediation of executive function (Li et al., 2019). Obesity, however, can lead to impaired executive function (Willeumier et al., 2011; Davidson et al., 2019). The results of this study may be explained by the leptin synthesized and secreted by adipocytes. Leptin is transported across the blood–brain barrier (BBB) *via* a saturable transport system (Banks, 2004) and is a potent modulator of excitatory synaptic transmission at hippocampal CA1 synapses (Irving and Harvey, 2014, 2021). Consequently, the ability of leptin to regulate excitatory synaptic efficacy at CA1 synapses suggests that leptin is likely to influence cognition processes (Hamilton and Harvey, 2021; Harvey, 2022). However, leptin transport across the BBB is impaired in high BMI phenotypes (Banks et al., 1999), which has an adverse effect on the synaptic transmission of hippocampal CA1 (Grillo et al., 2011). Furthermore, circulating leptin levels were significantly reduced in patients with cognitive impairment (Power et al., 2001; Johnston et al., 2014), which may explain why patients with SCD have increased CA1 gray matter volume and impaired executive function.

In this study, we did not find significant association between gray matter volume changes in CA1 and inhibitory control function. Previous studies have found that the impairment of executive function is not always correlated with the change of hippocampal subregion volume that is partly consistent with our results. Ge et al. (2021) showed that the volume of hippocampal subregions such as CA1 gradually decreased from the amyloid-negative group to the amyloid-positive group in the elderly people (including 87 individuals with normal cognition, 46 with MCI, and 10 with AD), and as amyloid pathology persisted, impairment of executive function was more significantly associated with changes in hippocampal tau lesions/volume. There seems to be a threshold effect in the relationship between hippocampal atrophy and executive function (Oosterman et al., 2012), that is, severe to very severe but not moderate hippocampal atrophy is associated with lower executive function. We speculate that due to the SCD in the preclinical stage of AD, its behavioral or brain pathological changes are not very serious, resulting in the weak correlation between executive function and hippocampal CA1 volume. Further study is needed to confirm this hypothesis.

This study has several limitations. First, the subjects we included did not contain any BMI level such as lower than 18.5 kg/m^2^, so it was impossible to examine the effects of insufficient body mass on executive function and hippocampal subregion. Secondly, the sample size included in this study is insufficient, which may lead to the instability of the research results. Furthermore, this is a cross-sectional study, and it is impossible to follow the subjects for a long time to observe the changes, correlations, and possible causes of cognitive function and hippocampal subregions caused by different BMI with the development of disease and age. Further longitudinal study should be done.

## Conclusion

Our results showed that higher BMI was associated with lower levels of executive function in the SCD and larger hippocampal subregion (CA1) gray matter volume. These associations suggest that obesity increases hippocampal gray matter volume in the elderly with SCD but is not sufficient to compensate for the impairment of executive function caused by the disease. Future studies are necessary to better elucidate these associations from the perspective of other mechanisms.

## Data availability statement

The raw data supporting the conclusions of this article will be made available by the authors, without undue reservation.

## Ethics statement

The studies involving human participants were reviewed and approved by Medical Ethics Committee of the Affiliated Rehabilitation Hospital of Fujian University of Traditional Chinese Medicine, Fuzhou, Fujian, China. The patients/participants provided their written informed consent to participate in this study. Written informed consent was obtained from the individual(s) for the publication of any potentially identifiable images or data included in this article.

## Author contributions

JLi: experimental design, analysis, and manuscript preparation and revision. RC: data analysis and manuscript preparation and revision. GC and SX: data collection and data analysis. QS and JLu: data collection. YW, ML, and HL: data analysis. All authors contributed to drafting the manuscript and have read and approved the final manuscript.

## Footnotes

1
https://www.softpedia.com/get/Science-CAD/MRIConvert.shtml

2
http://surfer.nmr.mgh.harvard.edu

3
https://surfer.nmr.mgh.harvard.edu/fswiki/HippocampalSubfieldsAn dNucleiOfAmygdala
