# Supplementary material for: Body mass index related to executive function and hippocampal subregion volume in subjective cognitive decline
Source: Front Aging Neurosci. 2022 Aug 17;14:905035. doi: 10.3389/fnagi.2022.905035 (PMC9428252; doi:10.3389/fnagi.2022.905035)
Supplement: Supplementary file 1 [file Data_Sheet_1.docx]

Supplementary Material

**Table 1. Consolidated table of hippocampal subregions (Iglesias et al., 2015)**

| **Before the merger** | **After the merger** |
| --- | --- |
| Hippocampal tail | Hippocampal tail |
| Hippocampal fissure | Hippocampal fissure |
| Fimbria | Fimbria |
| Parasubiculum | Parasubiculum |
| HATA | HATA |
| Subiculum head | Subiculum |
| Subiculum body |  |
| Presubiculum head | Presubiculum |
| Presubiculum body |  |
| CA1 head | CA1 |
| CA1 body |  |
| CA3 head | CA3 |
| CA3 body |  |
| CA4 head | CA4 |
| CA4 body |  |
| Molecular layer head | Molecular layer |
| Molecular layer body |  |
| GC-ML-DG head | GC-ML-DG |
| GC-ML-DG body |  |

HATA, hippocampal amygdala transition area; CA, cornu ammonis; GC-ML-DG, granule cell layer and molecular layer of the dentate gyrus.

**
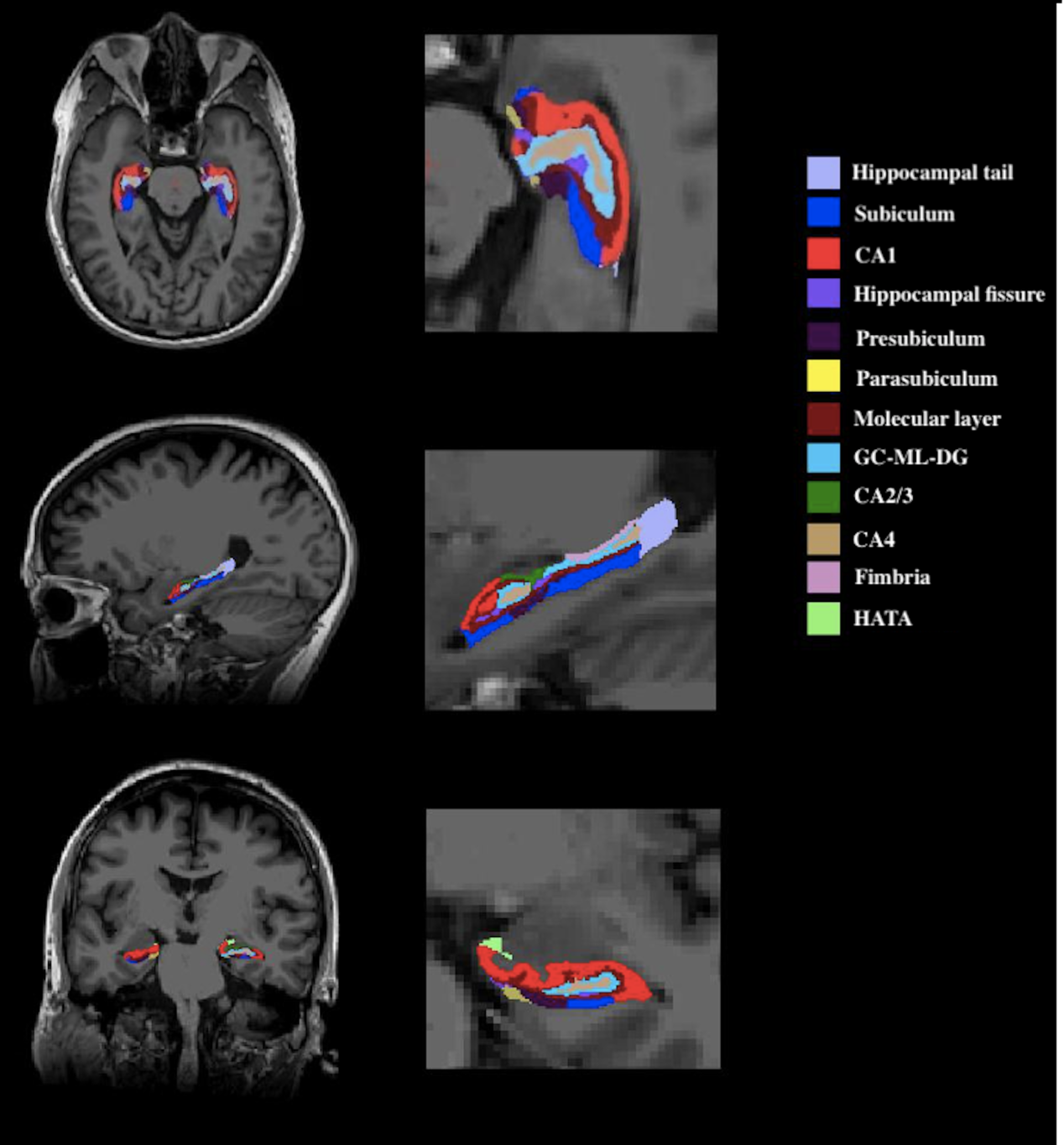
**

**Figure 1. Graphical representation of hippocampal subfields segmentation (Fischl, 2012)**

HATA: hippocampal amygdala transition area; CA: cornu ammonis; GC-ML-DG: granule cell layer and molecular layer of the dentate gyrus.

**Reference:**

Fischl, B. (2012). FreeSurfer. *NeuroImage* 62(2)**,** 774-781. doi: 10.1016/j.neuroimage.2012.01.021.

Iglesias, J.E., Augustinack, J.C., Nguyen, K., Player, C.M., Player, A., Wright, M., et al. (2015). A computational atlas of the hippocampal formation using ex vivo, ultra-high resolution MRI. *NeuroImage***,** 115-137.

**Table 2. The correlation between CA1 gray matter volume and SCWT C time(s)**

|  | **NC** | | **SCD** | |
| --- | --- | --- | --- | --- |
|  | **Normal BMI**  **（*n*=29）** | **High BMI**  **（*n*=17）** | **Normal BMI**  **（*n*=38）** | **High BMI**  **（*n*=27）** |
| ***r*** | 0.023 | 0.267 | -0.177 | -0.214 |
| ***P*** | 0.912 | 0.356 | 0.308 | 0.315 |

The partial correlation analysis was carried out with age, gender and years of education as covariates.

**Table 3. The correlation between CA1 gray matter volume and SCWT C-B time(s)**

|  | **NC** | | **SCD** | |
| --- | --- | --- | --- | --- |
|  | **Normal BMI**  **（*n*=29）** | **High BMI**  **（*n*=17）** | **Normal BMI**  **（*n*=38）** | **High BMI**  **（*n*=27）** |
| ***r*** | 0.050 | 0.261 | -0.191 | -0.169 |
| ***P*** | 0.807 | 0.367 | 0.273 | 0.430 |

The partial correlation analysis was carried out with age, gender and years of education as covariates.
